# Genetic Association of Peptidoglycan Recognition Protein Variants with Inflammatory Bowel Disease

**DOI:** 10.1371/journal.pone.0067393

**Published:** 2013-06-19

**Authors:** Fareeha Zulfiqar, Iztok Hozo, Sneha Rangarajan, Roy A. Mariuzza, Roman Dziarski, Dipika Gupta

**Affiliations:** 1 Indiana University School of Medicine–Northwest, Gary, Indiana, United States of America; 2 Department of Mathematics, Indiana University Northwest, Gary, Indiana, United States of America; 3 The Institute of Bioscience and Biotechnology Research, University of Maryland, Rockville, Maryland, United States of America; National Jewish Health and University of Colorado School of Medicine, United States of America

## Abstract

Inflammatory bowel disease (IBD) is a common disease, includes Crohn's disease (CD) and ulcerative colitis (UC), and is determined by altered gut bacterial populations and aberrant host immune response. Peptidoglycan recognition proteins (PGLYRP) are innate immunity bactericidal proteins expressed in the intestine. In mice, PGLYRPs modulate bacterial populations in the gut and sensitivity to experimentally induced UC. The role of PGLYRPs in humans with CD and/or UC has not been previously investigated. Here we tested the hypothesis that genetic variants in *PGLYRP1*, *PGLYRP2*, *PGLYRP3* and *PGLYRP4* genes associate with CD and/or UC and with gender and/or age of onset of disease in the patient population. We sequenced all *PGLYRP* exons in 372 CD patients, 77 UC patients, 265 population controls, 210 familial CD controls, and 24 familial UC controls, identified all polymorphisms in these populations, and analyzed the variants for significant association with CD and UC. We identified 16 polymorphisms in the four *PGLYRP* genes that significantly associated with CD, UC, and/or subgroups of patient populations. Of the 16, 5 significantly associated with both CD and UC, 6 with CD, and 5 with UC. 12 significant variants result in amino acid substitutions and based on structural modeling several of these missense variants may have structural and/or functional consequences for PGLYRP proteins. Our data demonstrate that genetic variants in *PGLYRP* genes associate with CD and UC and may provide a novel insight into the mechanism of pathogenesis of IBD.

## Introduction

Inflammatory bowel disease (IBD) is a common disease characterized by abdominal pain, diarrhea, malabsorption, and intestinal bleeding, and includes Crohn's disease (CD) and ulcerative colitis (UC). IBD is a complex multigenic and multifactorial disorder, and is due to an altered gut microbiome and an aberrant immune response [1]–[3]. Linkage and genome-wide association studies (GWAS) identified 163 susceptibility loci for IBD, out of which 110 are associated with both CD and UC, 30 with CD, and 23 with UC [4]–[9]. These GWAS studies have substantially increased our understanding of genetic factors that contribute to the susceptibility and pathogenesis of IBD, and they implicate a diverse array of genes that function in microbial recognition [10]–[12] and host immune responses [4]–[9], [13]–[16]. However, for the majority of these loci the causative genes and their precise role in the development and pathogenesis of IBD remain unknown. Furthermore, the majority of cases of heritable IBD have no identified genetic variation associated with the disease [17]. Thus, specific genetic and environmental factors that cause IBD still remain mostly unidentified.

**Figure 1:**
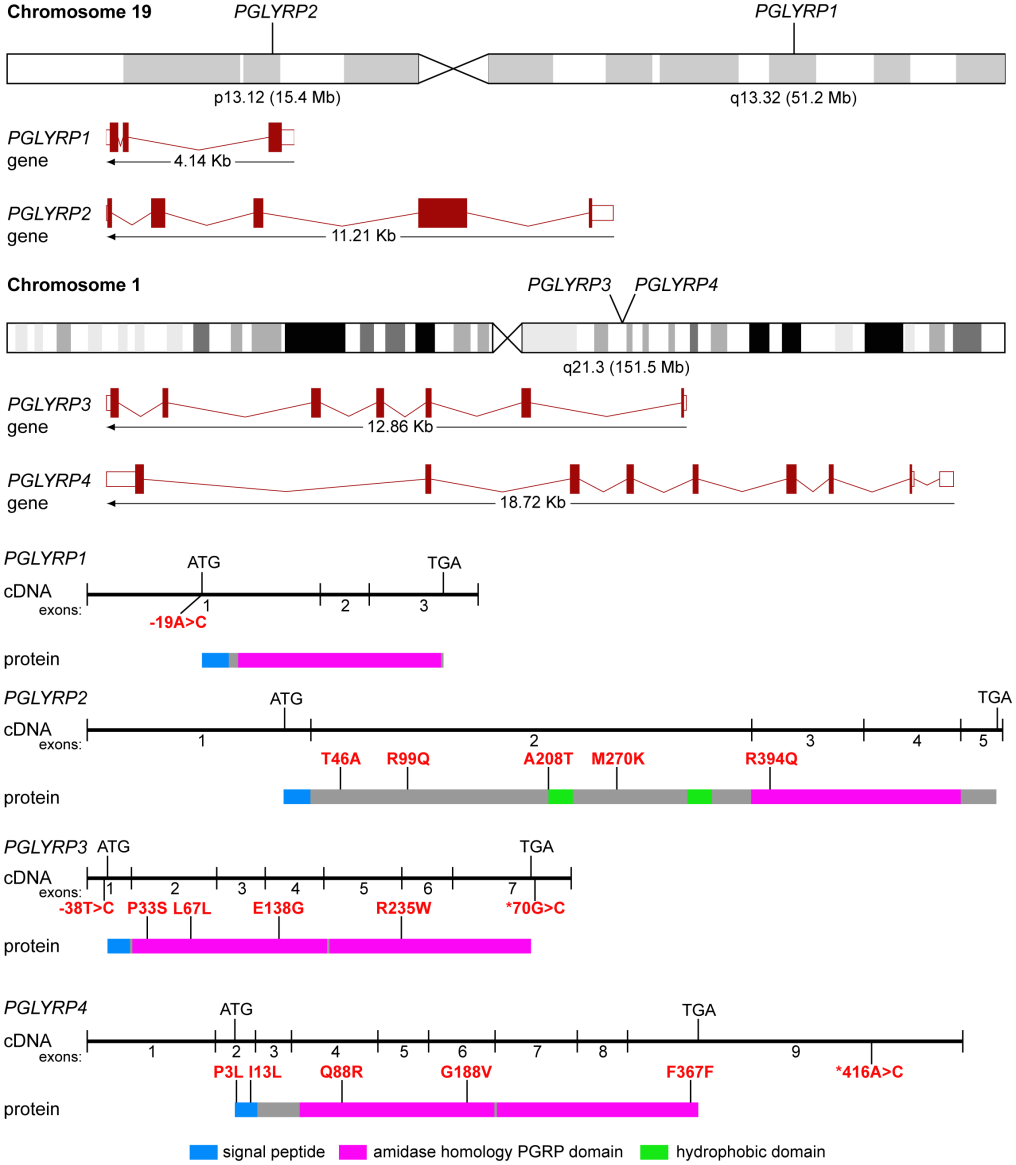
Chromosomal locations of *PGLYRPs* and schematic gene, cDNA, and protein structures, with positions of polymorphisms significantly associated with CD and/or UC. Genes show exons and introns, cDNAs show exons and start and stop codons, and proteins show domains and locations of significant polymorphisms.

Peptidoglycan recognition proteins (PGRPs or PGLYRPs) are innate immunity proteins that are conserved from insects to mammals and function in antibacterial immunity [18]–[20]. Mammals have four PGLYRPs, PGLYRP1, PGLYRP2, PGLYRP3, and PGLYRP4 [21], [22]. All PGLYRPs have one or two amidase/PGRP domains, which are homologous to the bacteriophage and bacterial type 2 amidases (Fig. 1) [18], [22], [23]. The amidase domain has peripheral α-helices and central β-sheets that form a cleft, which binds peptidoglycan, the essential and main component of bacterial cell wall [22]–[27]. In addition to binding peptidoglycan, PGLYRPs also bind other microbial cell wall components, including lipopolysaccharide, the main component of the outer membrane of Gram-negative bacteria [18], [23], [28]–[33]. PGLYRP1, PGLYRP3, and PGLYRP4 are bactericidal [28], [30] and kill bacteria by a novel mechanism: they over-activate bacterial stress-response two-component systems and induce lethal membrane depolarization and oxidative stress in bacteria [20]. PGLYRP2 is an N-acetylmuramoyl-L-alanine amidase that hydrolyzes bacterial peptidoglycan and is also bactericidal [34], [35].

All mammalian PGLYRPs are secreted proteins [18], [19]. PGLYRP1 is highly expressed in neutrophils and eosinophils and to a lower extent in epithelial and other cells [28], [29], [31], [36]. Other PGLYRPs are expressed in epithelial cells in the skin and mucous membranes, including intestinal tract, and PGLYRP2 is also expressed in the liver [22], [28], [37].

PGLYRPs are required to maintain a normal gut microbiome in mice, which protects mice from experimental ulcerative colitis. *Pglyrp1* ^−/−^, *Pglyrp2* ^−/−^, *Pglyrp3* ^−/−^, and *Pglyrp4* ^−/−^ mice are all more sensitive to dextran sulfate sodium (DSS)-induced colitis compared with wild type mice [37]. DSS-treated *Pglyrp*-deficient mice show greater loss of body weight, more severe intestinal bleeding, more severe colon pathology, higher production of IFN-γ, and increased number of NK cells in the colon compared with wild type mice [37]. In the absence of PGLYRPs the gut flora changes to a more damaging, proinflammatory microbiome and these changes are responsible for the increased sensitivity to colitis in *Pglyrp*-deficient mice [37].

PGLYRPs also modulate sensitivity to other inflammatory diseases in experimental animals. PGLYRP2 protects mice against psoriasis-like skin inflammation [38] and is required for the development of experimental arthritis [39], whereas PGLYRP3 and PGLYRP4 protect mice against atopic dermatitis [40]. By contrast, PGLYRP1 has a pro-inflammatory effect in three mouse models of inflammatory skin diseases (psoriasis, atopic dermatitis, and contact dermatitis) [38], [40] and in experimentally induced asthma [41], but has anti-inflammatory effect in experimentally induced arthritis [39]. Thus, each PGLYRP has a unique non-redundant effect, and for this reason PGLYRPs do not compensate for each other in mice deficient in a single PGLYRP, probably because of a non-redundant role of each PGLYRP in maintaining normal microbiome.

Peptidoglycan is the ligand for several pattern recognition molecules and is a potent stimulant of innate immunity [18], [19]. PGLYRP1 can inhibit some of these immunostimulatory activities by binding to peptidoglycan and preventing its recognition by innate immunity receptors [31], [33]. Hydrolysis of peptidoglycan by PGLYRP2 can also eliminate some of its immunostimulatory activities [19], [30]. However, these effects of PGLYRPs on peptidoglycan are mostly unrelated to their ability to modulate inflammation *in vivo*, because PGLYRPs modulate inflammatory responses induced not only by peptidoglycan [31], [33], [39], but also by unrelated allergens and antigens [37]–[40]. Therefore, modulation of inflammation by PGLYRPs is most likely indirect, through their effects on the microbiome.

Thus, the role of PGLYRPs in animal models of inflammatory diseases is well established, but the role of PGLYRPs in human diseases remains unknown, except for the previously reported association of *PGLYRP3* and *PGLYRP4* with psoriasis [42], [43]. The data from animal models suggest that PGLYRPs may also modulate the sensitivity to IBD and other inflammatory diseases in humans. In this study we hypothesized that genetic variants in *PGLYRP* genes may associate with IBD in humans. This hypothesis was based on (i) the chromosomal locations of *PGLYRPs*: *PGLYRP2* is located in the IBD susceptibility locus 19p13 [44]–[46] and *PGLYRP3* and *PGLYRP4* are located at 151.5 Mb on chromosome 1 near the 151.79 Mb IBD locus [9]; (ii) our data showing a role for PGLYRPs in experimental UC and other inflammatory diseases in mice [37]–[41]; and (iii) our data showing the effects of PGLYRPs on microbiome [37]. Here we tested our hypothesis by sequencing all exons of *PGLYRP1, PGLYRP2, PGLYRP3*, and *PGLYRP4* genes in CD and UC patients and disease-free controls, identifying all polymorphisms in *PGLYRP* genes, and analyzing these variants using tests of association. We also tested the hypothesis that different *PGLYRP* polymorphisms may associate with gender and/or with age of onset of disease. Our justification for doing these additional analyses was that IBD patients present with heterogeneous clinical manifestations and varied characteristics and responses to treatments. There are also gender differences in some clinical manifestations of CD and UC. This complex and varied presentation of the disease is most likely due to different combinations of causative genetic and environmental factors in different individuals. Moreover, the genetic profiles that contribute to IBD may be different for men and women, even if the overall susceptibility is not significantly different between genders. Our results demonstrate that polymorphisms in *PGLYRP1, PGLYRP2, PGLYRP3*, and *PGLYRP4* genes associate with susceptibility to CD and UC, and with gender and/or age of onset in both CD and UC patients. We also predict that several of the missense polymorphisms may have deleterious effects on the structure and/or function of PGLYRP proteins.

## Materials and Methods

### Ethics statement

This study was performed on DNA samples provided by the Crohn's and Colitis Foundation of America (CCFA) from their DNA data bank (www.ccfadatabank.org), in accordance with CCFA rules and regulations under the CCFA Material Transfer Agreement, approved by CCFA and Indiana University. DNA was isolated from cell lines that originated from anonymous donors de-identified by CCFA. This study did not involve recruitment of any human participants or collection of samples from any human participants and was deemed exempt by the Indiana University School of Medicine Institutional Review Board.

### DNA samples

All DNA samples from 372 CD patients, 77 UC patients, 210 familial CD controls, 24 UC familial controls, and 265 population controls (total number of samples 957) were obtained from the Crohn's and Colitis Foundation of America (CCFA, www.ccfadatabank.org). Population controls had no IBD, were not related to CD and UC patients, and were matched for age, race, and gender with the probands. All individuals within the above patient and population control groups were unrelated. In addition, 108 of the 372 CD patients had one or two familial unaffected controls (total number of controls 210) and 14 of the 77 UC patients had one or two familial unaffected controls (total number of controls 24). Familial controls include father and/or mother of the probands. To obtain DNA, blood samples were collected from probands and family controls at participating organizations (Massachusetts General Hospital, University of North Carolina, Mount Sinai, University of Chicago, Cleveland Clinic Foundation, Children's Hospital in Philadelphia, and University of Pittsburgh) and from population controls at Broad Institute. Immortalized cell lines were produced from blood cells, and DNA was extracted and purified from these cell lines by CCFA. All DNA samples were de-identified by CCFA and all the donors were anonymous.

### Amplification of exons and purification of amplified products

Primers to amplify all exons in *PGLYRP1* (3 exons), *PGLYRP2* (5 exons), *PGLYRP3* (7 exons), and *PGLYRP4* (9 exons) genes (Fig. 1) were designed using Primer3 software based on DNA sequence deposited in Ensembl. The Ensembl versions for genomic and mRNA that were used as reference sequences were: *PGLYRP1*, ENSG00000008438.3, ENST00000008938; *PGLYRP2*, ENSG00000161031.7, ENST00000340880; *PGLYRP3*, ENSG00000159527.3, ENST00000290722; and *PGLYRP4*, ENSG00000163218.10, ENST00000359650 respectively. All primers were purchased from InVitrogen. Primer sequences and the size of the amplicons are shown in Table S1. Some of the larger exons were amplified in more than one amplicon. Amplifications were performed in 10 µL reactions containing 10 ng genomic DNA, 200 nM each primer, 250 µM dNTPs, 2.5 mM magnesium chloride and 1.25 U Taq DNA polymerase in the standard PCR buffer (Promega). The PCR cycles included a denaturation step at 95°C for 6 minutes, followed by 40 cycles, each consisting of 94°C for 30 seconds, annealing at 56°C (or primer set–specific annealing temperature) for 30 seconds and 72°C for 30 seconds followed by extension for 7 minutes at 72°C. The PCR products were analyzed on 1.2% agarose gel and purified with 10 units Exonuclease I and 2 units FastAP™ Thermosensitive Alkaline Phosphatase (Fermentas) at 37°C for 15 minutes followed by incubation at 80°C for 15 minutes.

### Sequencing of amplified products

Primers used for amplification were also used for bidirectional sequencing of the amplified products. All sequencing was performed at the University of Chicago Comprehensive Cancer Center DNA sequencing and Genotyping Facility (Chicago) using the Sanger method on Applied Biosystems 3730XL 96-capillary and 3130 16-capillary automated DNA sequencers. Sequences were analyzed and polymorphisms were identified by Blast analysis against reference sequences deposited in NCBI. All sequencing chromatograms were also analyzed to definitively identify polymorphisms.

We selected the Sanger sequencing method instead of newer techniques such as single-stranded confirmation polymorphism, high performance liquid chromatography or massive parallel sequencing. The reasons for selecting the Sanger method are: it is highly reliable, reproducible, and specific, and provides detailed sequence information in a single step approach [47]. Furthermore, with the Sanger method a two-fold coverage is sufficient for molecular diagnostics, whereas massive parallel sequencing technologies typically require 20-fold coverage to confirm polymorphisms [47]. Often Sanger sequencing is used to confirm polymorphisms identified by other technologies [47].

In the first round of amplification, we sequenced and analyzed all exons in all four *PGLYRP* genes from 96 of the 372 CD samples and 96 of the 265 population control samples, and we identified 39 polymorphic markers. Subsequently, only those markers that were significant or approached significance in this initial study were tested in the remaining CD and population control samples and in the CD family controls. All 39 markers were tested in all UC and UC family controls samples. 35 of the 39 polymorphic markers were annotated in dbSNP and 4 were new. We submitted the 4 new SNPs to dbSNP (NCBI) and each SNP is assigned an identification number (SS#), which are: 647514526, 647514528, 647514529, and 647514530 (also listed in Tables S2 and S3).

### Statistical analyses

Each genetic marker was tested for Hardy Weinberg equilibrium in patient and control populations and markers that were not in equilibrium were excluded at *P*<0.05. The association of the specific markers between cases (CD or UC) and controls were tested using the Chi-square (χ^2^) test of independence and the Cochran Armitage Trend test for trend. The association of specific markers between case (CD or UC) and family controls was tested using the transmission disequilibrium test. These calculations were done in Excel using statistical formulas. Logistic regression analysis for combinations of polymorphisms was also performed using PLINK [48]. We analyzed the data by comparing the following groups: (a) all patients (CD or UC) with all controls, (b) all female patients (CD or UC) with control females, (c) all male patients (CD or UC) with control males, (d) CD female patients with CD male patients, and (e) UC female patients with UC male patients. CD and UC samples were subdivided into 3 groups based on age at diagnosis of the disease (in years): 5–20, 21–30, and ≥31, and the following groups were compared for CD and UC: (a) 5–20 compared with 21–30, (b) 5–20 compared with ≥31, and (c) 21–30 compared with ≥31. We performed additional analyses between males and females in these three age groups. Genetic polymorphisms that were significantly different in any of these comparisons (*P*≤0.05) were tested for strength of association by calculating the odds-ratio (OR) with 95% confidence intervals (CI).

### Structure analysis

The effect of missense polymorphisms on the structure and function of PGLYRPs were predicted using the SWISS-MODEL workspace [49]. The crystal structure of human PGLYRP3 (PGRP-Ialpha C-terminal; PDB, 2APH) was utilized as the template to generate the theoretical PGLYRP structures. Amino acid changes within the amidase/PGRP domain were analyzed by this method, however amino acid changes that are not in this domain could not be modeled because the crystal structure for these regions is not available. SWISS-MODEL repository is a database of annotated 3D protein structure models generated by the SWISS-MODEL homology-modeling pipeline. On the basis of a sequence alignment between the target protein and the template structure, three-dimensional models for the target protein were generated along with detailed template selection log, target-template alignment, summary of the model building and model quality assessment. The SWISS-MODEL database uses multiple model quality assessment tools to estimate the reliability of the resulting models [49]–[52].

## Results

### Overview of sequence variations in *PGLYRPs*

To identify variations in the *PGLYRP1, PGLYRP2, PGLYRP3 and PGLYRP4* genes that associate with CD and UC, we sequenced and analyzed all PGLYRP exons in a total of 957 DNA samples including 372 CD samples, 77 UC samples, 210 familial CD controls, 24 UC familial controls, and 265 population controls. CD patients were 91% of white European descent and 9% have African American, American Indian, Asian, or native Hawaiian descent. UC patients were 96% of white European descent and 4% have African American, American Indian, Asian, or native Hawaiian descent. Population controls were 90% of white European descent and 10% of African American, American Indian, Asian, or native Hawaiian descent. Information on gender and age of onset of disease is shown in Table 1.

**Table 1:** Demographic characteristics of the populations in this IBD study.

Patient subpopulation	Crohn's disease	Ulcerative colitis	Population controls
	*N* = 372	*N* = 77	*N* = 265
**Gender**	%	%	%
Male	45.4	59.7	52.1
Female	54.6	40.3	47.9
**Age at diagnosis (yrs)**	%	%	
5 to 20	41.9	26	
21 to 30	33.6	28.5	
≥31	24.5	45.5	
**Males and age of diagnosis (yrs)**	%	%	
5 to 20	48.5	21.7	
21 to 30	30.7	34.8	
≥31	20.8	43.5	
**Females and age of diagnosis (yrs)**	%	%	
5 to 20	38.7	32.3	
21 to 30	37.7	19.3	
≥31	23.6	48.4	
**Families (** ***N*** **)**	108	14	

Our analysis identified a total 39 polymorphisms in the exons of *PGLYRP1, PGLYRP2, PGLYRP3 and PGLYRP4* genes (Tables S2 and S3). All these variations were single nucleotide polymorphisms (SNP) with one exception, a 3 bp insertion in *PGLYRP4* (rs66641591). Amongst all SNPs there were more transitions (28/38, 74%) than transversions (10/38, 26%), a result consistent with other studies on genetic variations. 4 of the 39 polymorphisms identified in these populations are novel and were deposited in the SNP database (dbSNP, NCBI). Based on an allele frequency in any of the populations, polymorphisms were considered common (allele frequency >3) or rare (allele frequency ≤3). 7 of the total variations were unique to CD samples and were not present in the UC samples, whereas, 4 were unique to UC and were not present in the CD samples. All markers that were unique to either patient population were rare variants.

There were 21 missense polymorphisms (resulting in an amino acid change), 9 synonymous polymorphisms (no amino acid change), and 5 polymorphisms in the untranslated regions (UTR) (Tables S2 and S3). Thus, the total number of missense polymorphisms was more than double the number of synonymous polymorphisms and higher than typically observed. Furthermore, the ratio of missense to synonymous polymorphisms was substantially higher in *PGLYRP3* (8∶1, 89%) and *PGLYRP4* (7∶2, 78%), than typically observed.

We tested the hypothesis that genetic variations in *PGLYRP1, PGLYRP2, PGLYRP3*, and *PGLYRP4* genes associate with CD and/or UC. We also tested the hypothesis that different polymorphisms in these genes differentially associate between males and female, between patients with early, mid, or late age of onset of disease, and between male and female patients with early, mid, or late age of onset of disease. We also analyzed all 39 markers for familial association in CD and UC families using the transmission disequilibrium test. There were no significant differences identified with the transmission disequilibrium test, which indicated that these genetic changes in *PGLYRP* genes were not associated with familial transmission of CD or UC (data not shown). We performed tests of association for genotypes and alleles for all variants, calculated *P* (χ^2^) and odds ratio (OR), and performed haplotype analyses for all variants in each gene with *P*≤0.05 considered significant.

Analyses of these groups revealed that 16 out of the 39 polymorphisms in *PGLYRP1, PGLYRP2, PGLYRP3*, and *PGLYRP4* genes had significantly different distribution between disease and control groups and an additional 2 variants approached significance (Fig. 1, Table 2). 12 out of these 18 variants were missense (resulting in an amino acid change), 2 were synonymous polymorphisms (no amino acid change), and 4 were in UTR. The positions of all significant polymorphisms are indicated in Fig. 1. Of the 16 significant variants, 2 were significant in the entire UC population, 1 was significant with the entire CD population, and all 16 were significant in subgroups of patient populations divided by age of onset and/or gender. All variants significant in the entire UC or CD populations are described below, followed by descriptions of variants that are significant in patient populations divided into subgroups based on gender and/or age of onset of disease.

**Table 2:** Summary of PGLYRP variants that significantly associate with Crohn's disease and ulcerative colitis.

		Crohn's disease		Ulcerative colitis	
Gene	Change	*P* (χ^2^)	Odds Ratio	*P* (χ^2^)	Odds Ratio
*PGLYRP1*	5′ UTR	0.026	2.34	0.001	3.95
*PGLYRP2*	Thr46Ala	0.006	0.28	0.026	0.23
	Arg99Gln	0.023	0.36	0.026	0.23
	Ala208Thr			0.043	nc
	Met270Lys	0.013	0.65	0.026	0.23
	Arg394Gln	0.005	2.39		
*PGLYRP3*	5′ UTR	0.038	1.36	0.022	8
	Pro33Ser			0.009	nc
	Leu67Leu			0.002	14.4
	Glu138Gly			0.033	nc
	Arg235Trp			0.043	nc
	3′UTR	0.029	2.48		
*PGLYRP4*	Pro3Leu	0.039	1.85		
	Ile13Leu	*0.060*	0.16		
	Gln88Arg	0.043	2.68		
	Gly188Val	0.054	1.69		
	Phe367Phe	0.0005	6.60		
	3′UTR	*0.059*	0.16		

Complete data are shown in Tables 3 to 14 and S2 to S

*P* values showing trend towards significance are shown in italics; nc, not calculated.

### *PGLYRP1* exon 1, c.-19A>C, UTR and PGLYRP3 exon 2 c.201G>A Leu67 associate with UC, and *PGLYRP4* exon 9, c.1113C>T, p.Phe367 associates with CD

*PGLYRP1* c.-19A>C (SNP rs2072562) is present in the 5′ UTR of *PGLYRP1* gene (Fig. 1) and was significantly different between UC and controls (*P*_AA_ = 0.006, OR = 2.06) with a recessive trend (Tables 3, S3, and S4) and may be protective for all UC patients. *PGLYRP3* c.201G>A (SNP rs79540951) is synonymous for Leu67 (Fig. 1). The genotype and alleles were significantly different between UC and control populations in recessive model (*P*_GG_ = 0.002, OR = 14.4) (Tables 3, S3, and S10). *PGLYRP4* c.1113C>T (SNP rs41310915) is a synonymous polymorphism in exon 9 of *PGLYRP4* gene (Fig. 1) that was significantly different between CD patients and population control individuals in recessive and additive models (*P*_CC_ = 0.0005) and associated strongly with the disease (OR = 6.60, Tables 4, S2 and S14). These results indicate that variants in PGLYRP1 and PGLYRP3 associate with UC and one variant in PGLYRP4 associates with CD.

**Table 3:** *PGLYRP1* and *PGLYRP3 variants* associate with UC patients.

	UC versus controls		
	*P* (χ^2^)	OR (95% CI)	*P* (CA)*
*PGLYRP1* exon1, rs2072562, c.-19A>C, UTR			
AA	**0.006**	**2.06 [1.27–3.46]**	**0.006** *
A	**0.007**	**1.75 [1.16–2.64]**	
*PGLYRP3* exon 2, rs79540951, c.201G>A, p.Leu67			
GA	**0.002**	**14.4 [1.58–130]**	**0.002** *
G	**0.002**	**14.0 [1.55–126]**	

Complete data are shown in Tables S2, S3, S4, and S10.

*CA, Cochran Armitage Trend test data for the recessive model.

**Table 4:** *PGLYRP-4* variant associates with CD patients.

	CD versus controls		
	*P* (χ^2^)	OR (95% CI)	*P* (CA)*
Exon 9, rs41310915, c.1113C>T, Phe367			
CT	**0.0005**	**6.60 [1.98–22.1]**	**0.0005** *
T	**0.0005**	**6.40 [1.93–21.2]**	

Complete data are shown in Tables S2 and S14.

*CA, Cochran Armitage Trend test data for the recessive model.

c.-19A>C is in the 5′ UTR of *PGLYRP1* and may affect the rate of translation of the protein, whereas c.1113C>T is in 3′ UTR of *PGLYRP4* and may regulate stability of mRNA. The c.201G>A polymorphism in PGLYRP3 is synonymous for Leu67 and thus should not affect the structure and function of PGLYRP3, but may affect the stability or splicing of the mRNA. These three variants were also significant in our secondary analyses with subgroups of patient populations and are described below.

### *PGLYRP1* exon 1, c.-19A>C, UTR also associates with subgroups of UC and CD

*PGLYRP1* c.-19A>C rs2072562 was also significantly different between (i) CD females with early (5–20 yrs) and late (≥31 yrs) age of onset of disease in recessive model (*P*_AA_ = 0.026, OR = 2.34) and in UC females compared with control males in recessive and additive models (*P*_AA_ = 0.001, OR = 3.95) (Tables 5, S2, S3, and S4). Our data indicate that c.-19A>C in the UTR of *PGLYRP1* is differentially distributed between UC and controls, between UC females and control females, and between CD females and control females, and that this polymorphism appears to be protective for all UC patients and UC females and for CD females in the 5–20 years group for age of diagnosis.

**Table 5:** *PGLYRP1* exon 1, SNP rs2072562, c.-19A>C, UTR, associates with gender and age of onset in CD and with gender in UC patients.

	CD females 5–20 versus CD females ≥31			UC females versus control females		
	*P* (χ^2^)	OR (95% CI)	*P* (CA)*	*P* (χ^2^)	OR (95% CI)	*P* (CA)
AA	**0.026**	**2.34 [1.10–4.97]**	**0.026** *	**0.001**	**3.95 [1.64–9.53]**	**0.001** *
A	0.118	1.53 [0.89–2.63]		**0.006**	**2.62 [1.30–5.30]**	

Complete data are shown in Tables S2, S3, and S4.

*CA, Cochran Armitage Trend test data for the recessive model.

### *PGLYRP2* variants associate with gender and/or age of onset in CD and UC patients

**Figure 2:**
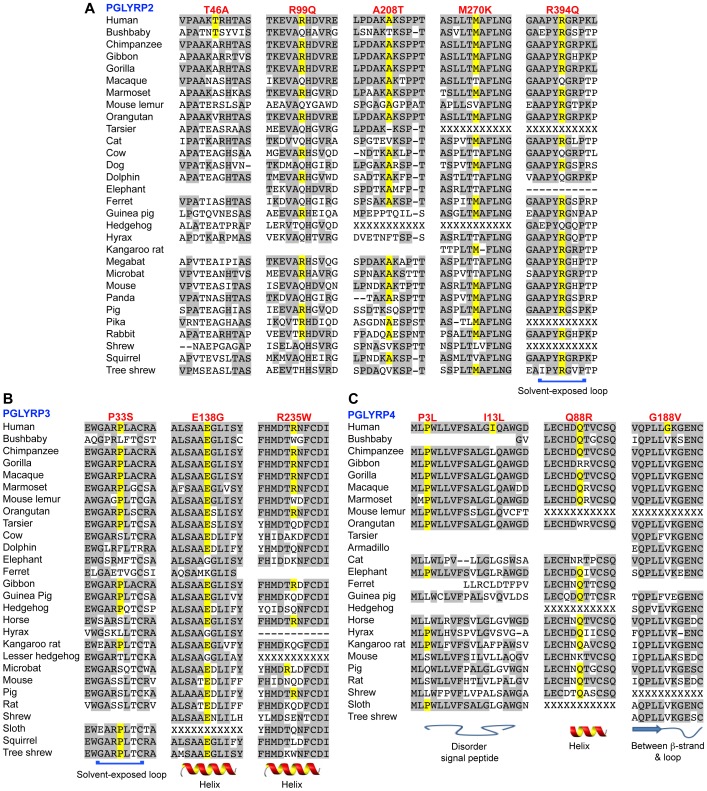
Amino acid sequence homology in mammalian PGLYRPs in the regions of non-synonymous polymorphisms significantly associated with CD and/or UC. Amino acids identical to human Ensembl reference sequences are shaded gray, conservation of the reference polymorphism is highlighted in yellow, and the predicted structure of the region is shown underneath the sequence. No sequence indicates truncated sequence, “–” indicates a gap in alignment, and “X” indicates incomplete sequence.

*Pglyrp2* c.136A>G (SNP rs3813135) is located in exon 2 and the frequency for this variant was significantly different in CD patients and CD female patients with early (5–20 yrs) and mid (21–30 yrs) age of onset of the disease in recessive and additive models (*P*_A_ = 0.026, OR = 2.17 and *P* = 0.006, OR = 3.57 respectively). The A>G variant was also significantly different in UC patients comparing age groups 5–20 vs. 21–30 (*P* = 0.026, OR = 0.23) (Tables 6, S2, S3, and S5). SNP rs3813135 is a missense variant that results in threonine to alanine substitution at position 46 in PGLYRP2 protein. Thr46 is located in exon 2 and is in the N-terminal region of PGLYRP2 (Fig. 1). The 3-D structure of this region of PGLYRP2 is not known and there is no homology model, thus the effect of the Thr46Ala was not predicted. Furthermore, because alanine is common in mammals (Fig. 2A), this substitution may be tolerated. Threonine has a hydroxyl group, which can serve as a site of attachment for oligosaccharide chains or for modification by phosphorylation. The loss of the hydroxyl group by an alanine substitution at this site may result in changes in posttranslational modifications. Our results indicate that c.136A>G variant in *PGLYRP2* is differentially distributed between early and mid age of onset of disease groups in all CD and UC patients and CD female patients and that the A>G polymorphism may increase the risk of developing CD or UC in the 21–30 age group.

**Table 6:** *PGLYRP2* exon 2 variants associate with gender and age of onset in CD and with age of onset in UC patients.

	CD 5–20 versus CD 21–30			CD females 5–20 versus 21–30			UC 5–20 versus UC 21–30		
	*P* (χ^2^)	OR (95% CI)	*P* (CA)*	*P* (χ^2^)	OR (95% CI)	*P* (CA)	*P* (χ^2^)	OR (95% CI)	*P* (CA)
rs3813135, c.136A>G, p.Thr46Ala									
AA or AG	0.170	2.01 [0.74–5.40]	**0.032** *	**0.010**	**5.81 [1.44–23.3]**	**0.005** *	**0.026**	**0.23 [0.06–0.86]**	0.288*
A	**0.026**	**2.17 [1.10–4.30]**		**0.006**	**3.57 [1.42–8.97]**		0.280	1.66 [0.66–4.17]	
rs733731, c.296G>A, p.Arg99Gln									
GG or GA	0.075	2.33 [0.91–5.98]	**0.046** *	**0.049**	3.88 [0.97–15.6]	**0.017** *	**0.026**	**0.23 [0.06–0.87]**	0.288*
G	**0.044**	**1.99 [1.01–3.89]**		**0.023**	**2.78 [1.14–6.78]**		0.280	1.66 [0.66–4.17]	

Complete data are shown in Tables S2, S3, S5, and S6.

*CA, Cochran Armitage Trend test data for the additive model.

c.296G>A (SNP rs733731) is located in exon 2 of *PGLYRP2* (Fig. 1). The G>A variant was significantly different between the groups for early (5–20 yrs) and mid (21–30 yrs) age of onset of the disease for all CD patients in a recessive model (*P* = 0.044, OR = 1.99), for CD females in recessive and additive models (*P* = 0.023, OR = 2.78) and for all UC patients (*P* = 0.026, OR = 0.231) (Tables 6, S2, S3, and S6). SNP rs733731 is a non-conservative missense variant that results in the substitution of arginine by glutamine at position 99 in PGLYRP2 protein (Fig. 1). Arginine is highly conserved in mammals (Fig. 2A) and the loss of a positively charged amino acid may potentially modify folding of the protein. However, the second most common amino acid at this position is glutamine (Fig. 2A), which may suggest that the arginine to glutamine substitution may be tolerated. Our results indicate that c.296G>A in *PGLYRP2* gene associates with CD, UC, and CD female patients in the mid age group for onset of the disease, and that the G>A variant may increase the risk for both CD and CD females in the 21–30 age group.

c.809T>A (SNP rs892145) is a common variant in *PGLYRP2* gene that was identified in all populations studied. The distribution of alleles was significantly different between CD females compared with CD males in a recessive model (*P* = 0.029, OR = 1.41) and compared with control females in recessive and additive models (*P* = 0.013, OR = 1.53) (Tables 7, S2, and S7). The T>A polymorphism was also significantly different in CD females (Tables S2, S3, and S7) and UC patients comparing the 5–20 and 21–30 age groups (Tables 7, S3, and S7). SNP rs892145 is a missense polymorphism, which results in a change from methionine to lysine at position 270 of PGLYRP2 protein (Fig. 1). Met270 is located in a region of the protein that is highly conserved in mammals and no mammalian PGLYRP2 has lysine at this position (Fig. 2A). Methionine has a nonpolar side chain, whereas lysine has a positively charged side chain, and based on SIFT analysis this variant is predicted to have a deleterious effect on PGLYRP2 (score 0.08). However, according to PolyPhen the variant is tolerated. Our results indicate that c.809T>A variant in *PGLYRP2* significantly associates with CD female patients and contributes to the risk factors for CD and UC in this subpopulation.

**Table 7:** *PGLYRP2* exon 2, SNP rs892145, c.809T>A, p.Met270Lys associates with gender and age of onset in CD patients.

	CD females versus CD males			CD females versus control females			UC 5–20 versus UC 21–30		
	*P* (χ^2^)	OR (95% CI)	*P* (CA)*	*P* (χ^2^)	OR (95% CI)	*P* (CA)	*P* (χ^2^)	OR (95% CI)	*P* (CA)
TT or TA	**0.024**	**0.62 [0.41–0.94]**	**0.024** *	**0.043**	**0.62 [0.40–0.98]**	0.015**	**0.026**	**0.23 [0.06–0.87]**	0.067*
A	**0.029**	**1.41 [1.04–1.91]**		**0.013**	**1.53 [1.10–2.11]**		0.506	1.13 [0.79–1.60]	

Complete data are shown in Tables S2, S3, and S7.

**Figure 3:**
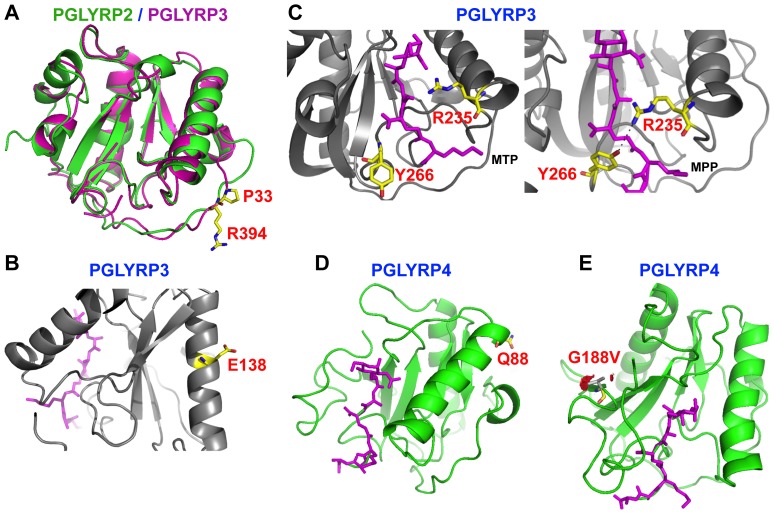
Location of missense polymorphisms in PGLYRPs that are predicted to impact the structure or function of amidase/PGRP domains. **A.** Superimposition of PGLYRP2 (green) and PGLYRP3 (magenta) structures shows similar locations of Arg394 (R394) in PGLYRP2 and Pro33 (P33) in PGLYRP3, highlighted in stick representation. Arg394 and Pro33 are located in a solvent-exposed loop of PGLYRP2 and PGLYRP3, respectively. The substitution Arg394Gln may alter folding of the protein or its interaction with the ligand. Proline adds structural rigidity in loops (due to it fixed torsion angle) and its mutation may increase flexibility of the loop and change the conformation of the protein. **B.** Mutation from Glu138 (E138) to glycine in PGLYRP3 may lead to loss of salt bridges and other interactions formed by glutamate in the α-helix and may affect protein stability. **C.** A comparison of the binding of peptidoglycan fragments, muramyl tripeptide (MTP, stick model in magenta, left image based on 1TWQ) with muramyl pentapeptide (MPP, stick model in magenta, right image based on 2APH) to the peptidoglycan-binding cleft in PGLYRP3. In the MTP-bound complex Tyr266 (Y266) faces away from MTP (left image), while the same residue turns in towards the peptide in the MPP-bound complex and functions to lock the peptide in the binding cleft (right image). Arg235 (R235) in the binding cleft interacts with several residues in the peptide through hydrogen bonds and van der Waals interactions along with Y266. A mutation of hydrophilic R235 to bulky hydrophobic Trp is predicted to alter these interactions and potentially affect binding of peptidoglycan. **D.** Gln88 (Q88) is located in the α-helix that forms a wall of the peptidoglycan-binding cleft in PGLYRP4. **E.** Location of Gly188 (yellow) and its mutation to valine in gray (G188V) in PGLYRP4 may lead to steric clashes with other residues, whereas substitution of valine with glycine would be tolerated. Location of the peptidoglycan fragment, MPP (stick model in magenta), in the binding cleft of PGRP domain is also shown in **D** and **E**.

* or **CA, Cochran Armitage Trend test data for the *recessive model or **additive model.

c.1181G>A (SNP rs4440547) in *PGLYRP2* gene is a common missense polymorphism that results in a non-conservative amino acid change, Arg394Gln. rs4440547 was significantly more frequent in CD females compared with CD males in a recessive model (*P* = 0.030, OR = 1.62) and in CD females compared with control females in recessive and additive models (*P* = 0.005, OR = 2.39) (Tables 8, S2, and S8). Arg394 is predicted to be located in a solvent exposed loop in the PGRP domain of PGLYRP2 (Figs. 2A and 3A) and is directly connected to a helix that forms a wall of the ligand binding cleft in PGLYRP2 (Fig. 3A). This region is highly conserved in mammals (Fig. 2A) and substitution of the positively charged arginine with the neutral residue glutamine could potentially affect protein folding and/or modify the affinity for peptidoglycan. Our data indicate that c.1181G>A in *PGLYRP2* significantly associates with CD females and may contribute to risk factors for CD in females.

**Table 8:** *PGLYRP2* exon 3, SNP rs4440547, c.1181G>A, p.Arg394Gln associates with gender in CD patients.

	CD females versus CD males			CD females versus control females		
	*P* (χ^2^)	OR (95% CI)	*P* (CA)*	*P* (χ^2^)	OR (95% CI)	*P* (CA)
GG+GA	**0.030**	**1.62 [1.05–2.51]**	**0.030** *	**0.005**	**2.39 [1.44–3.98]**	**0.004** *
A	**0.014**	**1.60 [1.10–2.31]**		**0.004**	**1.84 [1.20–2.83]**	

Complete data are shown in Tables S2 and S8.

*CA, Cochran Armitage Trend test data for the recessive model.

### *PGLYRP3* variants associate with gender and/or age of onset in CD and UC patients

c.-38T>C (SNP rs3006473) is present in the UTR of *PGLYRP3* gene (Fig. 1). The frequency of the T and C alleles was significantly different between CD females and CD males in a recessive model (*P* = 0.038, OR = 1.36). rs3006473 was also significant in UC females comparing the 5–20 with ≥31 age groups in a dominant model (*P* = 0.022, OR = 8) (Tables 9, S2, S3, and S9). Our data indicate that c.-38T>C polymorphism in *PGLYRP3* significantly associates with CD females and with UC females in the 5–20 age group.

**Table 9:** *PGLYRP3* exon 1, SNP rs3006473, c.-38T>C, UTR associates with gender in CD and with gender and age of onset in UC patients.

	CD females versus CD males			UC females 5–20 versus UC females ≥31		
	*P* (χ^2^)	OR (95% CI)	*P* (CA)*	*P* (χ^2^)	OR (95% CI)	*P* (CA)
TC	0.744	1.07 [0.71–1.61]	**0.041** *	**0.022**	**8.00 [1.21–52.7]**	0.022**
C	**0.038**	**1.36 [1.02–1.82]**		0.248	1.96 [0.62–6.19]	

Complete data are shown in Tables S2, S3, and S9.

* or **CA, Cochran Armitage Trend test data for the *recessive mode or **dominant model.

c.201G>A (SNP rs79540951) in *PGLYRP3* is synonymous for Leu67. The GG and GA genotypes were significantly different between UC and control populations (Table 3) and were also significantly different between UC females and control females in recessive and additive models (*P*_GG_ and *P*_GA_ = 0.0004) (Tables 10, S3, and S10). The A allele was not present in control females, thus the OR was not calculated. Our results indicate that c.201G>A variant in *PGLYRP3* significantly associates with UC and UC females. This polymorphism is synonymous for Leu67 and thus should not affect the structure and function of PGLYRP3, but may affect the stability or splicing of the mRNA.

**Table 10:** *PGLYRP3* exon 2, SNP rs79540951, c.201G>A, p.Leu67 associates with gender in UC.

	UC females versus control females		
	*P* (χ^2^)	OR (95% CI)	*P* (CA)*
GG	**0.0004**	not calculated	**0.0004** *
G	**0.0005**	not calculated	

Complete data are shown in Tables S3 and S10.

*CA, Cochran Armitage Trend test data for the recessive model.

c*70G>C (SNP rs2771110) is a common polymorphism in exon 7 of *PGLYRP3* (Fig. 1) and was present in all populations studied. The combined GG and GC genotypes were significantly different between CD patients with early (5–20) and late (≥31) age of onset of CD in a recessive trend (*P*_GG+GC_ = 0.016), and were associated with higher risk of disease in patients with an early onset of disease (OR = 1.91) (Tables 11, S2, and S11). The combined GC and CC genotype frequency for rs2771110 was also significantly higher in CD males with an early (5–20) onset of the disease compared with CD males with a late (≥31) age of onset of the disease in a recessive model (*P* = 0.029, OR = 2.48). Our results indicate that the c*70G>C variant associates with CD and specifically with CD males with an early onset of the disease (diagnosis in the age range of 5–20). SNP rs2771110 is located 70 bases downstream of the stop codon in the UTR of *PGLYRP3* (Fig. 1) and may modulate stability and half-life of *PGLYRP3* mRNA.

**Table 11:** *PGLYRP3* exon 7, SNP rs2771110, c*70G>C, UTR associates with age of onset and gender in CD patients.

	CD 5–20 versus CD≥31			CD males 5–20 versus CD males ≥31		
	*P* (χ^2^)	OR (95% CI)	*P* (CA)*	*P* (χ^2^)	OR (95% CI)	*P* (CA)
GG+CC	**0.016**	**1.91 [1.12–3.24]**	**0.016** *	**0.029**	**2.48 [1.09–5.69]**	**0.029** *
C	0.058	1.47 [0.99–2.18]		0.085	1.71 [0.93–3.15]	

Complete data are shown in Tables S2 and S11.

*CA, Cochran Armitage Trend test data for the recessive model.

### *PGLYRP4* variants associate with gender and/or age of onset in CD patients

*PGLYRP4* exon 2, c.8C>T (SNP rs12096209) was significantly different between CD females and CD males in recessive and additive models (*P* = 0.039, OR = 1.85) (Tables 12, S2, and S12). rs12096209 results in the amino acid substitution Pro3Leu. Pro3 is located in the signal peptide in PGLYRP4 (Fig. 1) and is highly conserved in mammalian PGLYRP4 (Fig. 2C). A change to leucine at this location may result in improper targeting of PGLYRP4. Our results indicate that c.8C>T polymorphism associates with UC males and may contribute to increased susceptibility to CD in males.

**Table 12:** *PGLYRP4* exon 2, SNP rs12096209, c.8C>T, p.Pro3Leu associates with gender in CD patients.

	CD females versus CD males		
	*P* (χ^2^)	OR (95% CI)	*P* (CA)*
CC	**0.039**	**1.85 [1.03–3.34]**	**0.039** *
C	**0.047**	**1.77 [1.01–3.11]**	

Complete data are shown in Tables S2 and S12.

*CA, Cochran Armitage Trend test data for the recessive model.

c.275A>G (SNP rs3006453) is a missense polymorphism in exon 4 of *PGLYRP4* gene (Fig. 1) and its distribution was significantly different in CD patients with an early onset of the disease (5–20 yrs) compared with CD patients with a late onset of the disease (≥31 yrs) in recessive and additive models (*P* = 0.043, OR = 2.68) (Tables 13, S2, and S13). rs3006453 was also significantly more frequent in CD males with early onset of disease (5–20 yrs) compared with CD males with a late onset of disease, (≥31 yrs, *P* = 0.018). The A>G polymorphism results in an amino acid substitution Gln88Arg. Gln88 is located on the α-helix that forms a wall of the predicted ligand binding cleft in PGLYRP4 (Fig. 3D) and is highly conserved in mammals (Fig. 2C). Introduction of a positive charge in place of this neutral amino acid may potentially affect the folding and/or stability of the protein. Our results indicate that *PGLYRP4* c.275A>G variant associates with all CD and CD males with early onset of the disease and that Gln88Arg in PGLYRP4 may contribute to the increased susceptibility to CD.

**Table 13:** *PGLYRP4* exon 4, SNP rs3006453, c.275A>G, p.Gln88Arg associates with age of onset and gender in CD patients.

	CD 5–20 versus CD≥31			CD males 5–20 versus CD males ≥31		
	*P* (χ^2^)	OR (95% CI)	*P* (CA)*	*P* (χ^2^)	OR (95% CI)	*P* (CA)
AA	**0.050**	0.37 [0.14–1.03]	**0.05**	**0.015**	not calculated	**0.015**
G	**0.043**	**2.68 [1.00–7.21]**		**0.018**	not calculated	

Complete data are shown in Tables S2 and S13.

*CA, Cochran Armitage Trend test data for the recessive model.

c.1113C>T (SNP rs41310915) is a synonymous polymorphism in exon 9 of *PGLYRP4* gene (Fig. 1) that was significantly different between CD patients and population control individuals (Table 4). The distribution of this polymorphism was also significantly different in CD females compared with control females (*P* = 0.0035) and the CT genotype strongly associated with the disease in females (OR = 11.3) (Tables 14, S2, and S14). These results indicate that SNP rs41310915 significantly associates with all CD patients and with CD female patients.

**Table 14:** *PGLYRP-4* exon 9, SNP rs41310915, c.1113C>T, Phe367 associates with gender in CD.

	CD females versus control females		
	*P* (χ^2^)	OR (95% CI)	*P* (CA)*
CT	**0.0035**	**11.3 [1.48–86.0]**	**0.0035**
T	**0.0040**	**10.8 [1.43–82.0]**	

Complete data are shown in Tables S2 and S14.

*CA, Cochran Armitage Trend test data for the recessive model.

### *PGLYRP2* p.Ala208Thr; *PGLYRP3* p.Pro33Ser, p.Glu138Gly, and p.Arg235Trp are rare or infrequent variants that associate with UC or with gender in UC patients

*PGLYRP2* exon 2, c.616G>A, p.Ala208Thr (Fig. 1) is a novel missense variant that was identified in one UC female (total *N* = 31) and was significantly different between UC and control groups and showed recessive and additive trends (*P* = 0.043, Table S15). Ala208 is highly conserved in primates (Fig. 2A) and the substitution of a nonpolar amino acid by a polar residue may disrupt interactions with other amino acids within PGLYRP2 or with other proteins.

*PGLYRP3* exon 2, c.97C>T SNP (rs150883037), p.Pro33Ser (Fig. 1) was significantly different between UC and control groups in recessive and additive models (*P* = 0.009, Table S15). Pro33 is highly conserved in mammals (Fig. 2B) and is located in a solvent exposed loop in the amidase/PGRP domain of PGLYRP3 (Figs. 2B and 3A). Proline residues provide rigidity to proteins due to the fixed torsion angel of the amino acid, which confers a physical limitation to the confirmation of the loop (Fig. 2). This loop is directly connected to a helix that forms a wall of the ligand binding cleft in PGLYRP3 (Fig. 3A). Interestingly, a second variant identified in this study, Arg394Gln, is located on a similar loop of PGLYRP2 and superimposition of the structures of PGLYRP 2 and PGLYRP3 demonstrates that Arg394Gln and Pro33Ser are spatially very close (Fig. 3A). A change in the flexibility of this loop could potentially affect protein-protein interactions, such as the proposed interaction of PGLYRP3 with proteins of bacterial two-component systems, which is thought to be essential for the bactericidal activity of PGLYRPs [19], [20].

*PGLYRP3* exon 4, c.413A>G (SNP rs41264632), p.Glu138Gly (Fig. 1) was significantly different between UC females and control females in recessive and additive models (*P* = 0.033, Table S15). c.413A>G results in the substitution of glutamate, a charged amino acid, with glycine, a nonpolar residue. Glu138 is highly conserved in mammals (Fig. 2B) and is located on an α-helix in the amidase/PGRP domain (Fig. 3B). Based on our *in silico* studies, a change from a bulky residue like glutamine to a small residue like glycine is predicted to result in a loss of salt bridges and hydrogen bonds that help stabilize the protein. Our results indicate that Glu138Gly associates with UC females and may result in destabilizing PGLYRP3 protein.

*PGLYRP3* exon 5, c.703C>T (SNP rs149128791), p.Arg235Trp (Fig. 1) was detected in one UC patient and was significantly different between UC females and control females in recessive and additive models (*P* = 0.043, Table S15). c.703C>T results in the amino acid change Arg235Trp in the amidase/PGRP domain (Fig. 1). Arg235 is highly conserved in primates (Fig. 2B) and is located in the ligand-binding cleft of PGLYRP3 and along with Tyr266 interacts with the ligand (Fig. 3C). Based on our *in silico* studies, a change from a hydrophilic residue to a bulky, hydrophobic residue, as in Arg235Trp is predicted to destroy these interactions and thus adversely affect the function of PGLYRP3.

### *PGLYRP4* exon 2, c.37A>C Ile13Leu, exon 6 c.575G>T Gly188Val, and exon 9, c.*416A>C, UTR variants may associate with CD or with gender in CD patients

*PGLYRP4* exon 2, c.37A>C, Ile13Leu (SNP rs30006458), was differentially distributed in CD females compared with CD males and the AA genotype was approaching significance (*P* = 0.06, Table S16). The predominant amino acid in the population is Leu13 (Table S5), which is highly conserved in mammals (Fig. 2C). Ile13 is located in the signal peptide of PGLYRP4 (Fig. 1) and may affect targeting of the protein.

*PGLYRP4* exon 6, c.575G>T, Gly188Val (SNP rs3006448) is a common polymorphism that was identified in all populations studied. The frequency of the GT genotype was significantly different in CD males compared with control males (*P* = 0.05), and approached significance in all CD patients (Table S16). For both comparisons, however, the OR 95% CI overlapped 1, indicating high variability. G is a minor allele at this site and T (coding for valine) is highly conserved in mammals (Fig. 2C). Gly188 is located in the amidase/PGRP domain (Fig. 1) in a highly conserved region (Figs. 2C and 3E), however as the predominant residue at position 188 in mammals is valine, the substitution to glycine may not be deleterious.

*PGLYRP4* exon 9, c.*416A>C (SNP rs2570440) was differentially distributed in CD females compared with males, and the AA genotype approached significance (P_A_ = 0.059, Table S16). Both variants showed a dominant trend. c.*416A>C is located in the 3′ UTR of PGLYRP4 mRNA (Fig. 1) and may affect the stability of the transcript.

### Variants in *PGLYRP2, PGLYRP3 and PGLYRP4* significantly associate with CD in haplotype groups

We next tested the association of multiple variants with CD and UC, based on haplotype analysis using a logistic regression model. All markers within a gene were grouped in all possible combinations and analyzed for association with CD and UC patients. Twelve different combinations of polymorphisms in *PGLYRP2* were significant in at least one comparison of CD patients with controls or within subgroups of CD patients. All variants in these combination groups are located in exon 2 of *PGLYRP2*. These twelve groups of markers were assigned numbers 1 through 12. The groups, markers and haplotypes that were significant are shown in Table S17. Three different combinations of polymorphisms in *PGLYRP3* and four different combinations of polymorphisms in *PGLYRP4* were significant in at least one comparison of CD patients with controls or within subgroups of CD patients. The groups, markers and haplotypes that were significant in *PGLYRP3* and *PGLYRP4* are shown in Table S18. Similar haplotype analyses were performed with UC patients, however no significant haplotypes were identified. These data further confirm our results demonstrating the association of genetic variants in *PGLYRP* genes with CD.

## Discussion

Our comprehensive analysis of the association of genetic polymorphisms in the exons of four *PGLYRP* genes demonstrates that *PGLYRP1*, *PGLYRP2*, *PGLYRP3*, and *PGLYRP4* are novel susceptibility genes for both CD and UC. We identified 16 polymorphisms that significantly associate with CD, UC, and/or subgroups of patients. Of the significant variants, 2 associate with the entire UC population, 1 with the entire CD population, and all 16 associate with gender and/or age of onset in the patient populations. We identified markers that uniquely associate with female or male populations or with patients with early, mid, or late onset of the disease. Of the 16 polymorphisms, 5 are common for CD and UC, 6 are unique for CD, and 5 are unique for UC (Table 2). An additional 2 polymorphisms approach significance and are associated with CD. Our haplotype analysis demonstrates combinations of variants in *PGLYRP2*, *PGLYRP3* and *PGLYRP4* genes that significantly associate with CD. 12 of these 16 variants result in an amino acid substitution, and 6 of these substitutions are in the amidase/PGRP domain and based on structural modeling we predict that some of these variants may change the structure and/or function of PGLYRPs.

*PGLYRP1* c.-19A>C, UTR and *PGLYRP3* c.201G>A Leu67Leu associate with the entire UC population and *PGLYRP4* c.1113C>T Phe367Phe associates with the entire CD population. The variant in *PGLYRP1* UTR may contribute to the susceptibility to UC by changes in protein expression for PGLYRP1. The synonymous variants may contribute to the disease by modulating stability of *PGLYRP3* or *PGLYRP4* mRNA. These three variants are also significant in our secondary analyses with subgroups of patient populations, which are discussed below.

The five polymorphisms that significantly associate with both CD and UC are: (i) *PGLYRP1*: c.-19A>C, UTR, (ii) *PGLYRP2*: Thr46Ala, Arg99Gln and Met270Lys and (iii) *PGLYRP3*: c.-38T>C, UTR The variant in *PGLYRP1* strongly associates with all UC patients and with UC females and moderately with CD females in the 5–20 age of diagnosis group. Thr46Ala and Arg99Gln variants in *PGLYRP2* associate with all CD, CD females, and UC female patients in the mid age group (21–30 yrs age of diagnosis). Met270Lys in PGLYRP2 associates with all CD females, and CD and UC females in the 5–20 age group. *PGLYRP3* c.-38T>C significantly associates with CD females and with UC females in the 5–20 age group. All three polymorphisms in *PGLYRP2* are in exon 2 and haplotypes including the three markers significantly associate with CD subgroups.

The six significant polymorphisms unique for CD patients are: (i) *PGLYRP2*: Arg394Gln; (ii) *PGLYRP3*: c*70G>C, UTR; and (iii) PGLYRP4: Pro3Leu, Gln88Arg, Gly188Val, and Phe367Phe. Variant Phe367Phe strongly associates with all CD and with CD females. SNP Pro3Leu associates with CD males. Three of these variants may further associate with CD females in the mid age range (21–30 yrs) for onset of disease. In contrast, c*70G>C in the 3′ UTR of PGLYRP3 and Gln88Arg in PGLYRP4 associate with CD patients with early onset of disease (5–20 yrs age of diagnosis).

The five significant polymorphisms unique for UC patients are: (i) *PGLYRP2*: Ala208Thr and (ii) *PGLYRP3*: Pro33Ser, Leu67Leu, Glu138Gly, and Arg23Trp. Thus, there are four missense variants in *PGLYRP3* that associate with UC and interestingly our previous data demonstrate that *Pglyrp3* ^−/−^ mice are highly sensitive to an experimental model of UC [37]. Variants Pro33Ser and Leu67Leu significantly associate with all UC samples, Ala208Thr, Leu67Leu, Glu138Gly, and Arg23Trp with UC females, and Pro33Ser with UC males. Ala208Thr and Arg235Trp are rare variants, each marker is present in one UC sample and analysis of a larger number of samples in the future will be necessary to validate these results. However, the association of rare variants with disease has recently been demonstrated for IBD [53] and for other diseases [54].

Four markers in *PGLYRP* genes significantly associate with all CD females and these are: (i) *PGLYRP2*: Met270Lys and Arg394Gln and (ii) *PGLYRP3*: c*70G>C, UTR and Phe367Phe. All markers are predicted to significantly increase the susceptibility to CD in females. Additional 3 markers became significant when the CD female samples are analyzed in subgroups based on the age of diagnosis and these are: (i) *PGLYRP1*: c.-19A>C UTR and (ii) *PGLYRP2*: Thr46Ala and Arg99Gln. The polymorphism in *PGLYRP1* is predicted to be protective for CD females in the early age of diagnosis group (5–20 yrs). In contrast, both missense mutations in *PGLYRP2* are predicted to increase susceptibility to CD in females in mid range of age of diagnosis (21–30 yrs). One marker significantly associates with CD males, Pro3Leu in *PGLYRP4*, and is predicted to increase susceptibility to CD in this sub-group. A different marker in the 3′ UTR of *PGLYRP3*, c*70G>C, is significant for CD males in the mid range (5–20) for age of diagnosis and is predicted to increase susceptibility to CD for this age group.

Twelve of the 16 significant polymorphisms result in an amino acid substitution and six of these variants are located in the amidase/PGRP domain. We predicted the effect of six variants on the protein structure and functions based on our previous crystal structures of the C-terminal PGRP domains of PGLYRP3 and PGLYRP4 [24]–[27]. All six residues are in highly conserved regions of PGLYRPs and non-conservative amino acid substitutions may have deleterious effects. Arg394 in *PGLYRP2* and Pro33 in *PGLYRP3* are both located in a solvent exposed loop of the proteins (Fig. 3A). The loss of a protein charge in the substitution Arg394Gln could affect protein folding and the affinity for the ligand. Pro33Ser substitution may increase the local flexibility of the loop and potentially alter interactions between PGLYRP3 and other proteins, such as members of the bacterial two-component systems, which are thought to be essential for the bactericidal activity of PGLYRPs [19,20]. The substitution Glu138Gly in PGLYRP3 is predicted to result in the loss of salt bridges and thus loss of protein stability (Fig. 3B). Arg235 in PGLYRP3 is located in the peptidoglycan-binding cleft and interacts with the ligand through hydrogen bonds and van der Waals forces (Fig. 3C). A substitution of the hydrophilic arginine with the bulky hydrophobic tryptophan is predicted to weaken the interactions between PGLYRP and its ligands and adversely affect the function of PGLYRP3. Gln88 in PGLYRP4 is part of the alpha helix that forms a wall of the peptidoglycan binding cleft (Fig. 3D), and the Gln88Arg substitution introduces a positively charged residue in place of a neutral residue and this change may affect the folding and stability to the protein.

Genetic variants in other proteins involved in microbe recognition and innate immune responses to bacteria are also associated with IBD. The NOD (nucleotide-binding oligomerization domain) proteins, NOD1 and NOD2, are similar to PGLYRPs in that they recognize bacteria through peptidoglycan fragments [55]–[57] and are required for maintaining homeostasis of the intestinal microbiome [58]. Though unlike PGLYRPs, NOD1 and NOD2 are cytosolic and binding of ligand initiates cell signaling and the release of inflammatory molecules [55]–[57]. Mutations in NOD2 are associated with Crohn's disease [10]–[12] and one of its major risk alleles increases risk for CD in heterozygous individuals by 2.4 fold and in homozygous or compound heterozygous individuals by 17.1 fold [12]. The mechanism of pathogenicity of NOD2 mutations for CD is thought to be an altered gut bacterial population, which results in chronic inflammation [59], [60]. By contrast to Nods, mammalian PGLYRPs are not signaling molecules, but rather secreted bactericidal proteins [18]–[20], [28], [30]. However, deletions of PGLYRPs also result in significant changes in microbiome, which are responsible for the increased susceptibility to experimental UC in mice [37]. Thus, we propose a similar mechanism for PGLYRPs: missense mutations in PGLYRPs could result in changes in bactericidal activity of the proteins, which could result in an altered gut microbiome followed by inflammation and increased susceptibility to IBD.

In conclusion, first, we have demonstrated that *PGLYRP1*, *PGLYRP2*, *PGLYRP3*, and *PGLYRP4* genes coding for bactericidal proteins associate with CD and UC, and with gender and/or age of onset in patient populations. Second, we have identified genetic markers in *PGLYRP* genes that associate with both CD and UC or are unique for CD or UC. Third, our study identifies potential causative variants that may alter the structure or function of PGLYRPs, thus, altering sensitivity to IBD. Identifying the mechanism by which PGLYRPs modulate sensitivity to IBD will require detailed studies focusing on the *in vitro* and *in vivo* effects of the variants identified in this study. Our results also support the polygenic nature of susceptibility of CD and UC, in which multiple genes contribute to the risk factors for acquiring the disease.

## Supporting Information

Table S1 ***PGLYRP*** **primers used in this study.**
(XLS)

Table S2 **Summary of** ***PGLYRP1*** **,** ***PGLYRP2, PGLYRP3*** **, and** ***PGLYRP4*** **variants in CD and case control cohorts.**
(XLS)

Table S3 **Summary of** ***PGLYRP1*** **,** ***PGLYRP2, PGLYRP3*** **, and** ***PGLYRP4*** **variants in UC and case control cohorts.**
(XLS)

Table S4 ***PGLYRP1*** **exon 1, SNP rs2072562, c.-19A>C, UTR, associates with UC and CD and with gender and age of onset in patients.**
(XLS)

Table S5 ***PGLYRP2*** **exon 2, SNP rs3813135, c.136A>G, p.Thr46Ala associates with gender and/or age of onset in CD and UC.**
(XLS)

Table S6 ***PGLYRP2*** **exon 2, SNP rs733731, c.296G>A, p.Arg99Gln associates with gender and/or age of onset in CD and UC.**
(XLS)

Table S7 ***PGLYRP2*** **exon 2, SNP rs892145, c.809T>A, p.Met270Lys associates with gender and/or age of onset in CD and UC.**
(XLS)

Table S8 ***PGLYRP2*** **exon 3, SNP rs4440547, c.1181G>A, p.Arg394Gln associates with gender and age of onset in CD.**
(XLS)

Table S9 ***PGLYRP3*** **exon 1, SNP rs3006473, c.-38T>C, UTR associates with gender and/or age of onset in CD and UC.**
(XLS)

Table S10 ***PGLYRP3*** **exon 2, SNP rs79540951, c.201G>A, p.Leu67 associates with UC and with gender in UC.**
(XLS)

Table S11 ***PGLYRP3*** exon 7, SNP rs2771110, c*70G>C, UTR associates with gender and/or age of onset in CD.
(XLS)

Table S12 ***PGLYRP4*** **exon 2, SNP rs12096209, c.8C>T, p.Pro3Leu associates with gender in CD.**
(XLS)

Table S13 ***PGLYRP4*** **exon 4, SNP rs3006453, c.275A>G, p.Gln88Arg associates with gender and/or age of onset in CD.**
(XLS)

Table S14 ***PGLYRP-4*** **exon 9, SNP rs41310915, c.1113C>T, Phe367 associates with CD and gender in CD.**
(XLS)

Table S15 **Association of rare or infrequent variants with UC or with gender in UC.**
(XLS)

Table S16 ***PGLYRP4*** **polymorphisms approaching significance for association with gender in CD.**
(XLS)

Table S17 **Significant haplotypes in** ***PGLYRP2*** **associate with CD in the following patients.**
(XLS)

Table S18 **Significant haplotypes in** ***PGLYRP3 and PGLYRP4*** **associate with CD in the following patients.**
(XLS)
